# Relating Remotely Sensed Optical Variability to Marine Benthic Biodiversity

**DOI:** 10.1371/journal.pone.0055624

**Published:** 2013-02-06

**Authors:** Kristjan Herkül, Jonne Kotta, Tiit Kutser, Ele Vahtmäe

**Affiliations:** Estonian Marine Institute, University of Tartu, Tallinn, Estonia; University of Western Australia, Australia

## Abstract

Biodiversity is important in maintaining ecosystem viability, and the availability of adequate biodiversity data is a prerequisite for the sustainable management of natural resources. As such, there is a clear need to map biodiversity at high spatial resolutions across large areas. Airborne and spaceborne optical remote sensing is a potential tool to provide such biodiversity data. The spectral variation hypothesis (SVH) predicts a positive correlation between spectral variability (SV) of a remotely sensed image and biodiversity. The SVH has only been tested on a few terrestrial plant communities. Our study is the first attempt to apply the SVH in the marine environment using hyperspectral imagery recorded by Compact Airborne Spectrographic Imager (CASI). All coverage-based diversity measures of benthic macrophytes and invertebrates showed low but statistically significant positive correlations with SV whereas the relationship between biomass-based diversity measures and SV were weak or lacking. The observed relationships did not vary with spatial scale. SV had the highest independent effect among predictor variables in the statistical models of coverage-derived total benthic species richness and Shannon index. Thus, the relevance of SVH in marine benthic habitats was proved and this forms a prerequisite for the future use of SV in benthic biodiversity assessments.

## Introduction

Biodiversity plays an important role in maintaining ecosystem integrity and services over long periods of time [1], [2], [3], [4], [5]. The cumulative effects of multiple human stressors such as resource extraction, pollution, habitat destruction, spread of non-indigenous species, and climate change have ever-increasing impacts on biological diversity. Mapping biodiversity at regional to global scales is increasingly important in order to sustainably manage natural resources and preserve biodiversity [6]. Time series of high resolution biodiversity maps can be used to detect change in natural systems and to assess the effects of management decisions on biodiversity patterns over space and time [7]. Mapping biodiversity by means of traditional sampling methods is expensive and time-consuming. Moreover, traditional sampling-point-wise field work is not suitable for covering extensive areas in high detail.

Rapid development of optical remote sensing techniques has opened a new avenue for seamless mapping of biodiversity over large areas. This has led to the development of new approaches to biodiversity measurements, including the use of indirect abiotic proxies, statistical modelling, remote sensing and a combination of the above [8], [9], [10], [11], [12]. These methods are of particular importance for marine biodiversity research as marine habitats are often hard to reach and the biota is relatively difficult to sample [13], [8], [12]. Nevertheless, existing studies have mainly focussed on predicting individual species distributions from physical variables (e.g. bathymetry derived measures, seabed sediment and hydrophysical properties of water) and, as such, have neglected biodiversity [14], [15], [16], [17], [18]. To date, remote sensing has seldom been used to directly map biodiversity in terrestrial ecosystems [19], [20], [21], [22] and has very rarely been used in the marine realm [8], [11].

Spaceborne and airborne optical remote sensing has the potential to quickly and cost-efficiently map large areas with high spatial resolution that would not be achievable with conventional *in situ* sampling. However, despite the advantages of remote sensing in marine ecosystems, *in situ* measurements remain the most important and reliable source of information on biodiversity. Combining field measurements, remote sensing, and statistical predictive models is currently seen as the most rewarding approach to mapping biodiversity at large spatial scales [10].

There are different approaches to examining relationships between remotely sensed optical parameters and biological diversity. Nagendra [23] proposed several ways: 1) direct mapping of species, an approach which is often unfeasible, 2) relating species occurrence to the remotely sensed habitat type, and, 3) correlating biological diversity with spectral radiance values. A further formulation of the third approach is known as the spectral variation hypothesis (SVH) [24], [25]. The SVH predicts a positive correlation between spectral variability (SV) of a remotely sensed image and biodiversity. As the water column absorbs a significant amount of bottom signal [26], optical remote sensing of aquatic diversity has not been as widely used as remote sensing of terrestrial diversity and SVH has only been tested on a few terrestrial plant communities [21], [22], [27].

Our study is the first attempt to apply the SVH in the marine environment. The aims of this study were: 1) to test the SVH in marine benthic macrophyte and macroinvertebrate diversity, and, 2) to assess the relative usefulness of SV and abiotic proxies (bathymetry derived measures, seabed sediment and wave exposure) for predicting benthic diversity.

## Materials and Methods

### 1. Study Area

**Figure 1:**
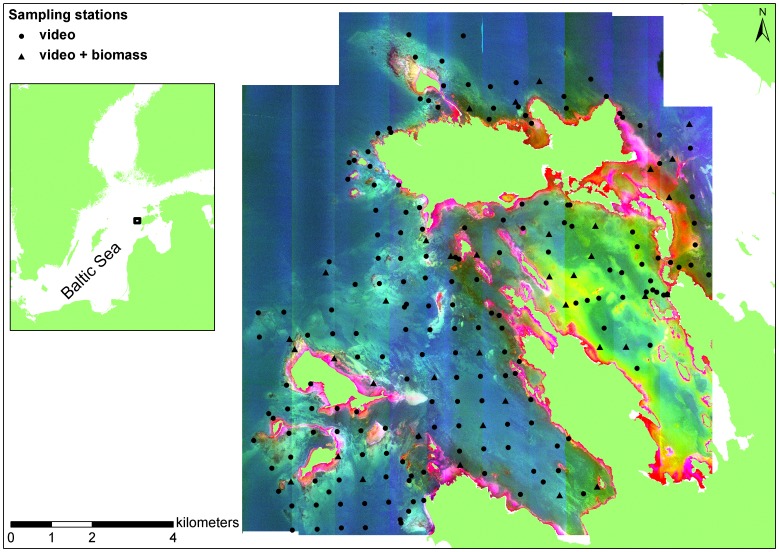
Study area. Filled circles and triangles indicate the location of sampling stations. Raster is the PCA image (3 first components) of CASI bands 5 to 16. PCA components 1, 2, and 3 are superimposed as red, green, and blue composite raster bands, respectively. PCA – principal component analysis; CASI – Compact Airborne Spectrographic Imager.

The study was conducted in coastal waters surrounding Saaremaa Island, in the eastern Baltic Sea (Figure 1). The area is characterized by complex topography with numerous islands, islets, bays, and peninsulas. The western part of the study area is wave-exposed while eastern bays are sheltered. Hard limestone substrate dominates in the exposed areas and soft silty sediments prevail in the sheltered bays. Different mixed sediments can be found in the mid-range of exposure gradient. The prevailing depth is 0.5−5 m with a maximum of about 10 m. Salinity ranges from 5 to 7.5. Regardless of low salinities, benthic flora and fauna are relatively diverse and abundant. Vascular plants and charophytes can be found at high densities in sheltered bays. Bladder wrack (*Fucus vesiculosus*) and several filamentous algae (e.g. *Ceramium tenuicorne*, *Polysiphonia fucoides*, *Cladophora glomerata*) dominate on hard substrate. Among invertebrates, the blue mussel (*Mytilus trossulus*) and the bay barnacle (*Amphibalanus improvisus*) prevail on hard bottoms; gammarid amphipods, idoteid isopods, the snails laver spire shell (*Hydrobia ulvae*) and river nerite (*Theodoxus fluviatilis*) are common in vegetated areas; and the lagoon cockle (*Cerastoderma glaucum*), the Baltic tellin (*Macoma balthica*) and the estuary ragworm (*Hediste diversicolor*) dominate in soft sediments.

### 2. Biological Sampling

Sampling was conducted in September 2010. A total of 207 sampling stations were visited (Figure 1). At each station the seabed was sampled by deploying a remote underwater video device from an anchored boat. The camera was set at an angle of 35° below horizontal and held 1 m above the sea floor resulting in a forward view of about 2 m. A full 360° rotation was captured at each station. All recorded videos were subsequently analysed by estimating the coverage of benthic macrophyte and invertebrate species. In addition to the video recordings, biomass samples were collected from 37 sampling stations. The sampling stations for biomass samples were located to maximize the coverage of different habitats. An Ekman type bottom grab sampler (0.02 m^2^) was used on soft sediment, while on hard surfaces scuba divers harvested all flora and fauna within a 0.04 m^2^ quadrat. Benthos samples were sieved through a 0.25 mm mesh and all retained material was transferred to plastic bags. The samples were stored deep frozen (–18°C) until analysis. In the laboratory, all samples were sorted under a binocular microscope (20–40 × magnification). All macrobenthic organisms were identified to species level except for oligochaetes, chironomids, and juveniles of gammarid amphipods (length <5 mm). Abundances and biomasses of all invertebrate taxa and biomasses of plant species were quantified. Prior to weighing, animals and plants were dried at 60°C for 48 hours and two weeks, respectively. Abundances and biomasses were calculated per square meter. Biomass sampling and analysis followed the guidelines developed for the HELCOM COMBINE programme [28] with the exception that no replicate samples were collected. The following biological diversity measures were used: number of benthic species/taxa, Shannon index (logarithm base e), and number of taxonomic and/or functional groups (“groups” hereafter). Macrophytes were divided according to their taxonomic belonging and included groups of green algae, brown algae, red algae, charophytes, and vascular plants. Macroinvertebrates were grouped according to their feeding mode and included groups of herbivores, deposit feeders, suspension feeders, and carnivores.

No specific permits are required for sampling benthos (invertebrates, plants) in Estonia. The collection of samples did not involve endangered or protected species. Estonian sea area is government property.

### 3. Remote Sensing

Airborne imagery was collected on September 1^st^ 2010 using hyperspectral imager CASI (Compact Airborne Spectrographic Imager; Itres, Canada) belonging to the Institute for Environmental Solutions, Latvia. The spectral range of the instrument is 370−1045.2 nm and widths of the spectral bands are programmable. We used 25 spectral bands (Table 1) located at wavelengths where different habitat types (sediment and biota) have distinct spectral features. The number and width of the bands were also optimized taking into account low water leaving signal and the speed of the aircraft. The aircraft was flown at an altitude of 2000 m resulting in a pixel size of 1 m. Flight direction was chosen taking into account the sun angle in order to minimize the sun and sky glint. Flight lines were planned in the form of ellipses shifting west from the previous path. In this way, a half of the study area was flown into the sun and a half of the study area off from the sun in order to minimise striped mosaic that may occur when flying back and forward. Pre-processing of the radiance imagery included cross-track illumination correction, geocorrection of the flight lines and mosaicking. The longitudinal extent of the mosaicked image was 11.6 km and latitudinal extent 12.9 km. Bands 1 to 4 and 17−25 were excluded as they were noisy and did not reflect seabed features. Individual bands of the CASI image were highly similar and principal component analysis (PCA) on bands 5 to 16 was used to reduce the amount of redundant information [29]. All further analyses were done using the three first principal components instead of original CASI bands.

**Table 1:** Central wavelengths of the CASI bands.

Band	Central wavelength (nm)
1	370.0
2	398.6
3	439.2
4	458.3
**5**	479.8
**6**	498.9
**7**	520.3
**8**	549.0
**9**	568.1
**10**	589.6
**11**	601.5
**12**	620.6
**13**	629.0
**14**	649.3
**15**	673.1
**16**	699.4
17	718.5
18	739.9
19	759.0
20	779.3
21	818.7
22	837.8
23	879.5
24	939.1
25	1045.2

Bands in boldface were used in the data analysis.

**Figure 2:**
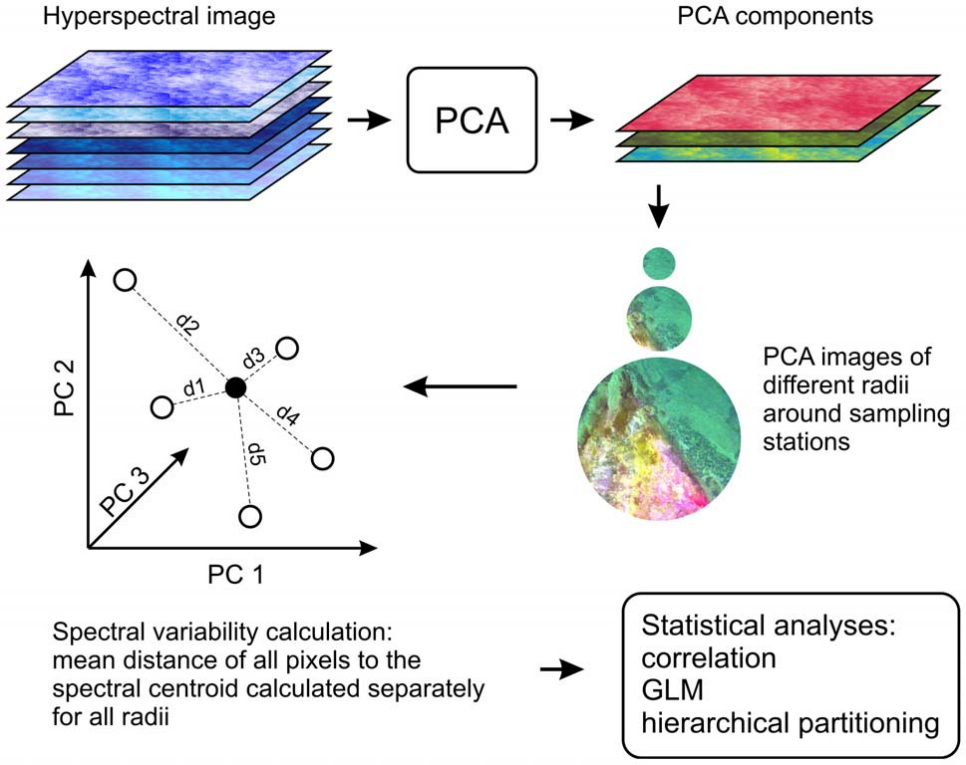
General flowchart of processing of the remotely sensed data.

Spectral variability (SV) was measured as a mean distance from spectral centroid of a given radius. The following radii were used: 5, 10, 15, 25, 50, 100, and 200 m. Spectral centroid was calculated as the mean value of each principal component in a specific radius. The distance of each pixel from the spectral centroid was then determined within each radius. The mean distance of all pixels from the spectral centroid in a given radius was considered as the SV of that radius. A general workflow of SV calculus is shown in Figure 2 (see Rocchini [21] and Oldeland et al. [22] for more detailed descriptions). The processing of CASI images and calculation of SV was done in the ESRI ArcInfo software.

### 4. Statistical Methods

Univariate regression was used to identify the correlation between CASI bands, CASI bands and principal components, spectral variability (SV) at different spatial scales, SV and biological variables. Generalized linear models (GLM) [30] were used to analyse the relationships between several environmental predictor variables and benthic biodiversity variables. Poisson distribution was used in the GLMs as it best suits for count data [30]. Models included depth, seabed slope, proportion of soft sediment, distance to land, distance to 10 m isobath, wave exposure [31], and SV as environmental predictor variables. SV at a scale that best correlated with the biological response variable was chosen for each model. Hierarchical partitioning [32] was used to identify the independent explanatory power of predictor variables in the GLMs. Hierarchical partitioning is an analytical method that enables quantification of the independent effects of all predictor variables on the response variable while handling problems of multicollinearity. The statistical significance of independent effects was obtained by using a randomization routine with 500 iterations [33]. All statistical analyses were done in the statistical software R version 2.11.1 [34].

## Results

### 1. CASI Imagery

All CASI bands were highly intercorrelated (Table 2) and the first three principal components cumulatively explained nearly 99% of the variance in CASI bands. The first principal component explained more than 96% of variance and had correlations of more than 0.9 with all the CASI bands. The second component had highest correlations with bands 15 and 16. The third component correlated the most with bands 9, 8, and 7 (Table 3).

**Table 2:** Correlations between the CASI bands.

	6	7	8	9	10	11	12	13	14	15	16
5	0.99	0.95	0.90	0.86	0.99	0.98	0.96	0.96	0.95	0.92	0.92
6		0.96	0.92	0.88	0.98	0.98	0.97	0.97	0.95	0.93	0.93
7			0.95	0.92	0.96	0.96	0.95	0.96	0.95	0.94	0.92
8				0.94	0.91	0.91	0.92	0.92	0.92	0.92	0.89
9					0.87	0.88	0.89	0.90	0.89	0.90	0.87
10						0.99	0.98	0.98	0.97	0.93	0.94
11							0.99	0.99	0.98	0.95	0.96
12								0.99	0.99	0.97	0.97
13									0.99	0.97	0.98
14										0.97	0.98
15											0.97

All correlations were statistically significant at p<0.05.

**Table 3:** Correlation of the CASI bands with the PCA components.

CASI band	PC1 (96.58)	PC2 (1.43)	PC3 (0.77)
5	0.98	0.15	−0.01
6	0.99	0.12	0.05
7	0.97	−0.01	0.20
8	0.93	−0.10	0.31
9	0.90	−0.15	0.35
10	0.99	0.09	−0.01
11	0.99	0.00	−0.04
12	0.99	−0.08	−0.03
13	0.99	−0.09	−0.02
14	0.99	−0.13	−0.03
15	0.96	−0.22	0.04
16	0.97	−0.21	−0.08

The proportion of variance in the CASI bands explained by the first three principal components are shown in brackets (%).

The values of spectral variability (SV) at different spatial scales were all statistically significantly intercorrelated with correlations being stronger between closer scales than distant scales (Table 4). SV at all spatial scales was significantly negatively correlated with depth, wave exposure and distance to land. All these correlations strengthened with increasing spatial scale (Table 5).

**Table 4:** Correlations between spectral variability at different spatial scales.

	10 m	15 m	25 m	50 m	100 m	200 m
5 m	0.90	0.81	0.70	0.62	0.57	0.54
10 m		0.96	0.87	0.78	0.69	0.63
15 m			0.96	0.85	0.73	0.65
25 m				0.91	0.78	0.69
50 m					0.94	0.81
100 m						0.91

All correlations were statistically significant at p<0.001.

**Table 5:** Correlations between spectral variability (SV) at different spatial scales and environmental variables.

SV	Depth	Prop. soft sediment	Wave exposure	Seabed slope	Distance to land	Distance to 10 m isobath
5 m	−**0.38**	−0.04	−**0.16**	0.19	−**0.30**	−0.06
10 m	−**0.42**	−0.05	−**0.17**	0.16	−**0.34**	−0.01
15 m	−**0.44**	−0.06	−**0.18**	0.16	−**0.37**	0.02
25 m	−**0.46**	−0.06	−**0.17**	0.16	−**0.39**	0.04
50 m	−**0.53**	−0.06	−**0.19**	0.17	−**0.44**	0.06
100 m	−**0.57**	−0.03	−**0.21**	0.20	−**0.48**	0.06
200 m	−**0.62**	−0.07	−**0.20**	0.25	−**0.55**	0.06

Statistically significant correlations at p<0.05 are shown in boldface.

### 2. Biological Data

A total of 17 macrophyte taxa and 4 macroinvertebrate species were identified in the video samples. The most common species (occurrence more than 30%) were *Mytilus trossulus*, followed by *Amphibalanus improvisus*, *Ceramium tenuicorne*, *Polysiphonia fucoides*, *Potamogeton pectinatus*, and *Fucus vesiculosus*. Based on video samples, macrophytes were present in almost 95% and macroinvertebrates in about 58% of sampling stations, respectively. See Table S1 for detailed data.

A total of 24 macrophyte species and 29 macroinvertebrate taxa were identified in the biomass samples. *Ceramium tenuicorne*, *Potamogeton pectinatus*, and *Polysiphonia fucoides* were the most frequently occurring macrophytes in the biomass samples. Among benthic invertebrates, *Cerastoderma glaucum*, *Theodoxus fluviatilis*, and *Hydrobia ulvae* were the most frequent. Macrophytes were found at 86% of biomass sampling stations and macroinvertebrates were found in every biomass sample. See Table S2 for detailed biomass data.

### 3. Correlations between SV and Biological Variables

#### Coverage data

The total number of macrobenthic species, number of macrophyte species and groups, and Shannon indices of macrobenthos and macrophytes were significantly positively correlated with SV at all spatial scales (Table 6). However, all the statistically significant correlations were weak with a maximum r of 0.32. The total number of macrobenthic species, number of macrophyte species, and Shannon indices were most strongly correlated with SV measured at a 10 m scale and the number of macrophyte groups with SV at a 200 m scale, respectively (Table 6).

**Table 6:** Pearson correlation coefficients between SV and biological variables at different spatial scales (m).

	Scale of SV (m)						
Biological variable	5	10	15	25	50	100	200
Coverage samples (n = 207)							
Macrophyte S	**0.25**	**0.31**	**0.28**	**0.25**	**0.26**	**0.26**	**0.30**
Total benthic S	**0.29**	**0.32**	**0.27**	**0.22**	**0.19**	**0.19**	**0.24**
Macrophyte groups	**0.26**	**0.28**	**0.27**	**0.24**	**0.26**	**0.26**	**0.31**
Macrophyte Shannon	**0.25**	**0.29**	**0.26**	**0.22**	**0.24**	**0.26**	**0.27**
Total Shannon	**0.22**	**0.24**	**0.20**	**0.15**	**0.14**	**0.16**	**0.19**
Biomass samples (n = 37)							
Macrophyte S	−0.06	−0.09	−0.04	0.01	0.08	0.12	0.13
Invertebrate S	0.25	0.26	0.23	0.22	0.28	0.32	0.32
Total benthic S	0.12	0.11	0.12	0.14	0.22	0.26	0.27
Macrophyte groups	−0.07	−0.04	0.04	0.07	0.11	0.08	0.07
Invertebrate groups	0.09	0.14	0.11	0.12	0.14	0.18	0.22
Macrophyte Shannon	−0.17	−0.17	−0.12	−0.08	−0.05	−0.06	−0.06
Invertebrate Shannon	0.17	0.12	0.14	0.16	0.21	0.20	0.21
Total Shannon	−0.19	−0.22	−0.23	−0.22	−0.22	−0.24	−0.18
Green algal S	−0.17	−0.16	−0.12	−0.05	0.03	0.07	0.13
Brown algal S	0.28	0.31	**0.37**	**0.39**	**0.46**	**0.46**	**0.38**
Red algal S	0.10	0.01	0.02	0.04	0.02	0.01	−0.01
Vascular plants S	−0.29	−0.32	−**0.33**	−**0.32**	−0.29	−0.24	−0.18
Herbivores S	**0.36**	0.32	0.32	0.31	**0.37**	**0.43**	**0.43**
Suspension feeders S	−0.14	−0.21	−0.28	−0.29	−0.25	−0.22	−0.12
Deposit feeders S	0.07	0.15	0.09	0.03	−0.01	−0.02	−0.09
Carnivores S	0.07	0.12	0.16	0.22	0.32	**0.32**	0.32

Statistically significant correlations at p<0.05 are shown in boldface. S – species richness.

#### Biomass data

Only the number of brown algal species, vascular plant species, herbivore species and carnivore species had statistically significant correlations with SV at some of the spatial scales (Table 6). The number of brown algal species, herbivore species, and carnivore species positively correlated with SV whereas the number of vascular plants negatively correlated with SV. The correlations between SV and biomass-based diversity variables reached up to 0.46 (Table 6).

### 4. Statistical Models and Effects of Predictor Variables

SV had statistically significant independent effect in all models that were based on coverage data but in only two models that were based on biomass data (Table 7). SV had the highest contribution among predictor variables in the models of total benthic species richness and total benthic Shannon index, both based on the coverage samples.

**Table 7:** Results of generalized linear models.

	Independent effects of predictor variables (%)								
Biological response variable	SV	Depth	Soft sed.	Wave exp.	Slope	Dist. land	Dist. 10 m	SV scale	Expl. dev.
Coverage samples (n = 207)									
Total benthic S	**34.98**	**10.76**	**30.04**	3.14	2.24	**18.39**	0.45	10	17.06
Macrophyte S	**15.30**	**28.36**	2.61	**11.57**	2.24	**22.39**	**17.54**	10	25.09
Macrophyte gr.	**10.34**	**31.03**	**10.84**	**12.32**	2.46	**19.21**	**13.79**	200	26.15
Total Shannon	**36.84**	5.26	**31.58**	5.26	2.63	**15.79**	2.63	10	11.15
Macroph. Shan.	**21.79**	**15.38**	3.85	**32.05**	1.28	**8.97**	**16.67**	10	22.71
Biomass samples (n = 37)									
Total benthic S	11.40	24.27	3.22	6.73	3.80	**49.42**	1.17	200	22.85
Macrophyte S	11.17	27.18	1.46	9.71	1.94	32.04	16.50	200	25.88
Invertebrate S	18.15	12.36	5.41	19.31	5.41	33.20	6.18	100	23.13
Macroph. gr.	4.04	21.21	19.19	16.16	3.03	16.16	20.20	50	24.38
Invertebr. gr.	7.81	**34.38**	7.81	28.13	1.56	12.50	7.81	200	27.41
Total Shan.	22.50	7.50	**42.50**	2.50	17.50	2.50	5.00	100	25.82
Macroph. Shan	12.50	21.88	6.25	15.63	**31.25**	6.25	6.25	10	27.11
Invertebr. Shan.	5.71	31.43	11.43	17.14	5.71	25.71	2.86	50	21.38
Green algal S	1.43	**20.57**	2.86	**41.43**	10.29	5.14	**18.29**	5	60.41
Brown algal S	22.12	11.50	**30.97**	1.77	7.08	**24.78**	1.77	50	51.44
Red algal S	2.44	29.27	9.76	2.44	19.51	26.83	9.76	5	22.73
Vasc. plants S	22.03	16.95	**20.34**	9.32	6.78	0.85	**23.73**	15	44.20
Herbivores S	**24.83**	15.86	1.03	**28.97**	4.48	16.55	8.28	100	36.87
Susp. feed. S	**24.39**	2.44	2.44	**37.80**	2.44	12.20	18.29	25	33.08
Deposit feed. S	24.30	15.89	28.04	1.87	7.48	1.87	20.56	10	28.63
Carnivores S	12.20	25.61	3.66	23.17	3.66	29.27	2.44	100	28.72

Independent effects of predictor variables calculated by hierarchical partitioning are shown with statistically significant effects (*p*<0.05) in boldface. SV at the spatial scale (SV scale) that most strongly correlated with the biological response variable (see Table 6) was used in each model. Expl. dev. – explained deviance (%) of a model, S – species richness, Shan. – Shannon index, gr. – number of groups.

## Discussion

The spectral variation hypothesis (SVH) [24], [25] predicts a positive correlation between spectral variability (SV) of a remotely sensed image and biodiversity. However, to date the SVH has been tested only on a few terrestrial plant communities [21], [22]. In this study we applied the SVH for the first time in the marine environment. The study demonstrated that all coverage-based benthic diversity measures had statistically significant positive correlations with SV indicating the relevance of SVH in marine benthic habitats. The relevance of SVH in marine benthic habitat forms a prerequisite for the future use of SV in benthic biodiversity assessments. The potential of SV in improving traditional methods of spatial modelling was further substantiated by the results of the GLMs where SV had the highest independent effect amongst environmental predictor variables in the models of benthic species richness and Shannon index of macrobenthos (both based on coverage data). This indicates that SV has a higher potential in applications of predicting more general diversity measures than diversity within a specific taxonomic or functional group. Intuitively, spectral signal variability is greater at the scales of whole-community or landscape, which include a multitude of optically differing species compared with spectral variability within a specific taxonomic group where species are optically more similar [35], [36], [37].

Regardless of the statistical significance of the correlations between benthic diversity measures and SV, the strength of the correlations was low. As water column absorbs a significant amount of bottom signal [38], [26], the strength of correlation between SV and biodiversity is expected to be higher in terrestrial environments than in aquatic environments. Rocchini [21] used imagery from four different satellites and found correlations of 0.4 to 0.7 between SV and species richness of a wetland. Oldeland et al. [22] used an airborne hyperspectral spectrometer and found correlations up to about 0.7 between SV and species richness in a savannah vegetation. In contrast, the correlations presented in our marine ecosystem ranged between 0.2 and 0.4. Palmer et al. [25] also found weak correlation between SV and species richness of grassland ecosystem, but they used a panchromatic aerial photograph as an input for SV calculation that captures considerably less optical variability compared to a hyperspectral sensor. Additionally, Palmer et al. [25] discuss inaccuracies in georeferencing and differences between the timing of image and sampling as possible reasons for weak correlations in their study.

Besides the absorbing effect of the water column there may be other reasons why the relationship between SV and biodiversity was found weaker in this study than in previous terrestrial studies. Our design included only one biological sample per one estimate of SV while Rocchini [21] pooled lists of species from four separate biological samples per one estimate of SV. Pooling diversity data from a higher number of samples per spatial unit of SV would have very likely resulted in significantly stronger correlations between biodiversity and SV in our study. Nevertheless, the emergence of statistically significant positive correlations with only one sample per unit of SV is even a stronger evidence of the validity of SVH in a marine environment than emergence of the effect when using several pooled samples.

Another reason that possibly contributed to the weak correlations was that the number of species that can be visually identified from video samples was limited to large and easily distinguishable species and the actual species diversity remained unrecorded. However, these large species were also the main contributors to the optical signal recorded by the airborne spectrometer. It is possible to identify all macrobenthic species from the biomass samples but due to the time constraints the number of collected samples is often low. Increasing the number of biomass samples may result in stronger correlations and higher numbers of statistically significant correlations.

Earlier studies have shown that the strength of relationships between SV and biodiversity vary with spatial scales, sites, diversity measures used, and imagery type [20], [21], [22]. Palmer et al. [25] suggested that the strength of correlation between SV and biodiversity increases with increasing spatial scale. Oldeland et al. [22] found this to be true in savannah plant diversity. Our analyses did not reveal strong overall effects of spatial scale of SV on biological variables. All coverage-derived diversity measures were statistically significantly correlated with SV at every scale without a clear effect of scale on the strength of correlation. However, it must be noted, that in our study, biological sampling was always done at the scale of a single sampling station while SV was estimated at different radii around the biological sampling point. Contrastingly, Oldeland et al. [22] had two different scales for biological sampling. The selection of spatial scales of estimates of SV ranged from 5 to 200 m in this study. Scales larger than 200 m should be included in future studies to further clarify scale-dependent relationships between SV and benthic diversity. Together with larger spatial scales, higher numbers of biological samples per unit of SV should be collected.

In contrast to coverage-based diversity variables, only a few diversity measures calculated from the biomass samples were statistically significantly correlated with SV. We suggest two explanations for this pattern. Firstly, as the number of biomass samples was much lower than the number of coverage estimates, the statistical power of analyses of biomass-derived measures was lower compared to the analyses of coverage-derived diversity measures. Secondly, the nature of coverage estimates, i.e. the visual information from the seabed, directly related to the optical signal received by the hyperspectral sensor while data from biomass samples included a lot of information not perceivable by remote optical means. This included the presence of species with small dimensions and very low biomasses, taxonomically close and optically similar species that were distinguished based on morphological (microscopic) features, and infaunal species. However, a positive relationship between visually perceivable and visually unperceivable heterogeneity can be expected because the former reflects the “landscape” for the latter.

Amongst diversity measures derived from biomass samples the species richness of brown algal species had the strongest correlation with SV. This may have been influenced by the fact that the depth distribution of brown algae is the widest among macrophyte groups studied [39], providing larger gradients in environmental variability that, in turn, may facilitate the emergence of stronger relationships between SV and brown algal species richness.

Species richness of vascular plants was the only variable that had statistically significant negative correlations with SV. Vascular plants predominate in sheltered to moderately exposed areas with homogenous soft seabed sediments where they may form dense beds [40]. These beds may consist of multiple species (*Potamogeton* spp, *Myriophyllum spicatum*, *Zannichellia palustris*) but due to their spectral similarity and the homogeneity of the substrate the SV of these areas are low and this may contribute to the negative relationship between vascular plant species richness and SV.

Species richness of herbivores was the only zoobenthic variable that was statistically significantly correlated with SV. Many previous studies have shown that herbivores are selective to algal species [41], [42] and they respond strongly to the amount of available resource [43] as plants provide habitat and food resources for herbivores [44]. Consequently, the diversity, biomasses of aquatic plants and thus, SV are expected to be positively correlated with the diversity of herbivores in many waterbodies [45], [46].

To conclude, the relevance of the SVH in marine benthic habitats was proved for the first time and this forms a basis for the further use of SV in benthic biodiversity assessments. The potential of SV in biodiversity assessments was further justified by the results of statistical models showing that SV often had the highest independent explanatory power amongst environmental variables to predict benthic biodiversity. This study also highlights the importance of biological sampling at multiple spatial scales in future studies in order to clarify scale-dependent relationships between SV and benthic diversity. The broader importance of the study lies in promoting our understanding of the patterns of macroalgal and invertebrate diversity in the coastal seascapes.

## Supporting Information

Table S1 **Occurrences and coverages of benthic species that were identified in video samples.**
(DOC)

Table S2 **Occurrences and biomasses of benthic species that were identified in biomass samples.**
(DOC)
