# Supplementary material for: Relating Remotely Sensed Optical Variability to Marine Benthic Biodiversity
Source: PLoS One. 2013 Feb 6;8(2):e55624. doi: 10.1371/journal.pone.0055624 (PMC3566085; doi:10.1371/journal.pone.0055624)
Supplement: Table S1 — Occurrences and coverages of benthic species that were identified in video samples. (DOC) [file pone.0055624.s001.doc]

| Species | Count | Occurrence (%) | Maximum coverage (%) | Mean cove­rage over all stations (%) | Mean cove­rage in stations of occurrence (%) |
| --- | --- | --- | --- | --- | --- |
| Macrophytes | | | | | |
| *Ceramium tenuicorne* | 78 | 37.68 | 65 | 4.89 | 12.97 |
| *Chara* sp. | 33 | 15.94 | 100 | 7.03 | 44.12 |
| *Cladophora glomerata* | 61 | 29.47 | 100 | 6.30 | 21.38 |
| *Fucus vesiculosus* | 70 | 33.82 | 100 | 11.58 | 34.26 |
| *Furcellaria lumbricalis* | 10 | 4.83 | 30 | 0.76 | 15.70 |
| *Monostroma balticum* | 1 | 0.48 | 1 | 0.00 | 1.00 |
| *Myriophyllum spicatum* | 12 | 5.80 | 20 | 0.39 | 6.75 |
| *Pilayella littoralis* | 16 | 7.73 | 15 | 0.37 | 4.75 |
| *Polysiphonia fucoides* | 75 | 36.23 | 75 | 4.61 | 12.72 |
| *Potamogeton pectinatus* | 72 | 34.78 | 100 | 9.95 | 28.60 |
| *Potamogeton perfoliatus* | 7 | 3.38 | 5 | 0.11 | 3.29 |
| *Ranunculus baudotii* | 7 | 3.38 | 35 | 0.28 | 8.29 |
| *Ruppia maritima* | 47 | 22.71 | 45 | 2.82 | 12.40 |
| *Sphacelaria arctica* | 5 | 2.42 | 5 | 0.06 | 2.60 |
| *Ulva intestinalis* | 1 | 0.48 | 25 | 0.12 | 25.00 |
| *Zannichellia palustris* | 3 | 1.45 | 5 | 0.03 | 2.33 |
| *Zostera marina* | 28 | 13.53 | 70 | 4.22 | 31.18 |
| Macroinvertebrates | | | | | |
| *Amphibalanus improvisus* | 95 | 45.89 | 10 | 1.19 | 2.59 |
| *Cordylophora caspia* | 1 | 0.48 | 1 | 0.00 | 1.00 |
| *Mytilus trossulus* | 120 | 57.97 | 60 | 4.92 | 8.49 |
| *Theodoxus fluviatilis* | 3 | 1.45 | 5 | 0.07 | 5.00 |
