# Supplementary material for: Relating Remotely Sensed Optical Variability to Marine Benthic Biodiversity
Source: PLoS One. 2013 Feb 6;8(2):e55624. doi: 10.1371/journal.pone.0055624 (PMC3566085; doi:10.1371/journal.pone.0055624)
Supplement: Table S2 — Occurrences and biomasses of benthic species that were identified in biomass samples. (DOC) [file pone.0055624.s002.doc]

| Taxon | Count | Occur­rence (%) | Maximum biomass (g m^-2^) | Mean biomass over all stations (g m^-2^) | Mean biomass in stations of occurrence (g m^-2^) |
| --- | --- | --- | --- | --- | --- |
| Macrophytes | | | | | |
| *Chaetomorpha linum* | 7 | 18.92 | 0.86 | 0.04 | 0.19 |
| *Chara aspera* | 9 | 24.32 | 194.71 | 22.46 | 92.34 |
| *Chara baltica* | 2 | 5.41 | 1.60 | 0.06 | 1.09 |
| *Chara canescens* | 4 | 10.81 | 30.41 | 1.99 | 18.38 |
| *Chara connivens* | 3 | 8.11 | 1.24 | 0.05 | 0.63 |
| *Chara horrida* | 5 | 13.51 | 24.28 | 1.07 | 7.88 |
| *Cladophora glomerata* | 13 | 35.14 | 275.36 | 22.40 | 63.75 |
| *Cladophora rupestris* | 4 | 10.81 | 23.50 | 0.84 | 7.74 |
| *Monostroma balticum* | 2 | 5.41 | 29.30 | 0.88 | 16.34 |
| *Ulva intestinalis* | 5 | 13.51 | 2.71 | 0.11 | 0.80 |
| *Ceramium tenuicorne* | 17 | 45.95 | 81.02 | 4.74 | 10.33 |
| *Ceramium virgatum* | 1 | 2.70 | 0.15 | 0.00 | 0.15 |
| *Coccotylus truncatus* | 2 | 5.41 | 1.40 | 0.05 | 0.84 |
| *Furcellaria lumbricalis* | 9 | 24.32 | 185.76 | 7.12 | 29.26 |
| *Polysiphonia fucoides* | 15 | 40.54 | 87.04 | 5.53 | 13.63 |
| *Rhodomela confervoides* | 2 | 5.41 | 1.06 | 0.04 | 0.75 |
| *Fucus radicans* | 1 | 2.70 | 2514.97 | 67.97 | 2514.97 |
| *Fucus vesiculosus* | 11 | 29.73 | 3015.33 | 213.67 | 718.72 |
| *Pilayella littoralis* | 7 | 18.92 | 67.32 | 2.65 | 14.02 |
| *Stictyosiphon tortilis* | 1 | 2.70 | 0.03 | 0.00 | 0.03 |
| *Myriophyllum spicatum* | 1 | 2.70 | 1.17 | 0.03 | 1.17 |
| *Potamogeton pectinatus* | 15 | 40.54 | 407.15 | 22.08 | 54.47 |
| *Zannichellia palustris* | 2 | 5.41 | 0.63 | 0.02 | 0.46 |
| *Zostera marina* | 7 | 18.92 | 132.68 | 6.27 | 33.16 |
| Macroinvertebrates | | | | | |
| *Gammarus* juv. | 22 | 59.46 | 0.74 | 0.07 | 0.12 |
| *Gammarus locusta* | 4 | 10.81 | 0.23 | 0.01 | 0.14 |
| *Gammarus oceanicus* | 6 | 16.22 | 5.29 | 0.32 | 2.00 |
| *Gammarus salinus* | 6 | 16.22 | 1.24 | 0.06 | 0.36 |
| *Gammarus tigrinus* | 9 | 24.32 | 7.94 | 0.52 | 2.15 |
| *Gammarus zaddachi* | 2 | 5.41 | 0.17 | 0.01 | 0.16 |
| *Idotea* sp. | 2 | 5.41 | 1.39 | 0.04 | 0.73 |
| *Idotea balthica* | 12 | 32.43 | 3.28 | 0.45 | 1.38 |
| *Idotea chelipes* | 6 | 16.22 | 3.16 | 0.16 | 1.02 |
| *Asellus aquaticus* | 2 | 5.41 | 0.47 | 0.02 | 0.35 |
| *Hydrobia ulvae* | 28 | 75.68 | 14.25 | 2.25 | 2.97 |
| *Jaera albifrons* | 4 | 10.81 | 0.04 | 0.00 | 0.02 |
| *Lymnaea peregra* | 12 | 32.43 | 66.46 | 2.77 | 8.54 |
| *Palaemon adspersus* | 1 | 2.70 | 18.23 | 0.49 | 18.23 |
| *Theodoxus fluviatilis* | 29 | 78.38 | 59.62 | 6.18 | 7.88 |
| *Amphibalanus improvisus* | 2 | 5.41 | 1.52 | 0.07 | 1.29 |
| *Cerastoderma glaucum* | 31 | 83.78 | 62.58 | 13.59 | 16.22 |
| *Mytilus trossulus* | 18 | 48.65 | 290.50 | 15.36 | 31.58 |
| *Chironomidae* | 20 | 54.05 | 3.91 | 0.36 | 0.67 |
| *Corophium volutator* | 3 | 8.11 | 0.04 | 0.00 | 0.02 |
| *Hediste diversicolor* | 13 | 35.14 | 1.02 | 0.07 | 0.19 |
| *Leptocheirus pilosus* | 1 | 2.70 | 0.03 | 0.00 | 0.03 |
| *Macoma balthica* | 12 | 32.43 | 21.96 | 1.89 | 5.83 |
| *Oligochaeta* | 3 | 8.11 | 0.02 | 0.00 | 0.01 |
| *Coleoptera* | 4 | 10.81 | 0.17 | 0.01 | 0.10 |
| *Crangon crangon* | 1 | 2.70 | 11.43 | 0.31 | 11.43 |
| *Cyanophthalma obscura* | 6 | 16.22 | 0.20 | 0.01 | 0.08 |
| *Lepidoptera* | 12 | 32.43 | 0.20 | 0.02 | 0.07 |
| *Odonata* | 1 | 2.70 | 0.40 | 0.01 | 0.40 |
| *Saduria entomon* | 2 | 5.41 | 2.30 | 0.07 | 1.36 |
| *Trichoptera* | 5 | 13.51 | 3.93 | 0.23 | 1.67 |
